# Tetanus Surveillance — United States, 2009–2023

**DOI:** 10.15585/mmwr.ss7501a1

**Published:** 2026-04-16

**Authors:** Michelle M. Hughes, Avnika B. Amin, Amy B. Rubis

**Affiliations:** ^1^Division of Bacterial Diseases, National Center for Immunization and Respiratory Diseases, CDC; ^2^U.S. Public Health Service Commissioned Corps; ^3^Epidemic Intelligence Service, CDC

## Abstract

**Problem/Condition:**

Tetanus is a serious but vaccine-preventable disease caused by the toxin produced by germinated spores of *Clostridium tetani* bacteria. Despite substantial declines in incidence resulting from immunization, cases continue to occur, particularly among unvaccinated and undervaccinated populations.

**Period Covered:**

2009–2023.

**Description of System:**

The National Notifiable Diseases Surveillance System uses national surveillance to identify cases of tetanus using the Council of State and Territorial Epidemiologists’ tetanus case definition. Tetanus cases identified through clinical diagnosis are reported to CDC by state health departments. Detailed tetanus-specific case information is requested, which includes tetanus toxoid–containing vaccine (TTCV) vaccination history, wound history, medical care before tetanus disease onset, and the clinical course of illness, including death.

**Results:**

During 2009–2023, a total of 402 tetanus cases and 37 associated deaths were reported from 47 states and the District of Columbia, with a mean annual tetanus incidence of 0.08 cases and 0.008 deaths per 1 million population. More than half (62.2%) of all reported tetanus cases occurred in males. Incidence was higher among males than females for all persons aged <65 years and higher among women than men for adults aged ≥80 years. Women aged ≥80 years had the highest overall tetanus incidence (0.27 cases per 1 million population). The overall case-fatality rate among persons with tetanus with known vital status was 12.4% (37 of 299), with deaths predominantly affecting older adults. A total of 45.0% of persons with tetanus who had a substantial wound sought medical care before disease onset. Among patients with wounds eligible for tetanus post-exposure prophylaxis, 2.3% received tetanus immune globulin (TIG) and 26% received TTCV per recommendations. Among persons whose vaccination history was known, a substantial proportion (43.9%) had not received any TTCV doses, highlighting substantial gaps in coverage.

**Interpretation:**

Despite being preventable through vaccination, tetanus continues to occur among persons of all age groups in the United States. Overall, males have higher incidence compared with females; the highest incidence is among older women. Approximately 1 in 10 persons who develop tetanus will die, with the highest mortality and case-fatality rates among older adults.

**Public Health Action:**

Multiple efforts might reduce the incidence of tetanus, including clinicians’ assessing for and offering routine tetanus vaccination for children and decennial tetanus boosters for adults. In addition, persons with significant wounds should seek timely medical care, and clinicians should provide recommended wound care, including identifying tetanus-prone wounds and the need for and administration of TTCV and TIG. Because *C. tetani* is ubiquitous in the environment, continued surveillance and vaccination efforts are crucial to monitor trends, identify opportunities to prevent tetanus cases, and reduce tetanus incidence in the United States.

## Introduction

Tetanus is a serious vaccine-preventable disease caused by the toxin produced by *Clostridium tetani*, a spore-forming bacterium that is ubiquitous in the environment (*1*). Tetanus is not transmitted person-to-person; exposure usually occurs through a contaminated or deep wound. Once inside the body in anaerobic conditions, *C. tetani* spores germinate and produce tetanospasmin (tetanus toxin), which is responsible for the serious effects of infection. The three clinical types of tetanus are 1) cephalic (least common), 2) localized, and 3) generalized (most common); neonatal tetanus is a form of generalized tetanus. Cephalic tetanus is associated with head and neck injuries and is characterized by cranial nerve palsies. Localized tetanus results in spasms confined to the area surrounding the site of injury. Generalized tetanus symptoms can include difficulty swallowing or breathing, generalized spasms, rigidity, seizures, and trismus (lockjaw). In the generalized and localized forms, the tetanus toxin affects the sympathetic nervous system by interfering with the release of neurotransmitters leading to unopposed muscle contractions and spasms. The incubation period can range from 1 to 21 days, with a longer incubation the farther the injury is from the central nervous system. Complications can include aspiration pneumonia, bone fractures resulting from spasms, hypertension, laryngospasm, nosocomial infection, pulmonary embolism, and death. The clinical course of tetanus is highly variable, with the acute phase generally lasting 1–4 weeks; for those who survive, recovery can take months. Tetanus toxin irreversibly binds to nerve terminals, so recovery depends on the creation of new neuromuscular connections and degradation of the toxin (*1*). Neonatal tetanus can occur when a mother is unvaccinated or undervaccinated against tetanus (precluding the passive transplacental transfer of antibodies which could protect the infant), especially in the context of nonsterile delivery conditions and umbilical cord care.

Tetanus is a clinical syndrome diagnosed on the basis of a clinical presentation consistent with tetanus in the absence of a more likely cause. No diagnostic tests exist that can support or rule out the diagnosis of tetanus. Tetanus has been a nationally notifiable disease since 1947. The numbers of tetanus cases and deaths have declined substantially since the introduction of the tetanus vaccine into the routine childhood immunization program during the late 1940s and as a decennial booster dose for persons of all ages during the 1960s (*2*,*3*). Since 1947, reported tetanus cases have declined >95%, and U.S. deaths from tetanus have declined >99%. However, cases continue to occur, particularly among persons who are unvaccinated or undervaccinated against tetanus (*4*,*5*). National surveillance for tetanus is conducted to monitor trends in incidence and identify populations at increased risk for morbidity and mortality. A previous surveillance summary reported on tetanus epidemiology in the United States during 2001–2008. This report provides an updated summary of tetanus surveillance in the United States during 2009–2023 (*6*).

## Methods

### Case Ascertainment and Surveillance Case Definition

CDC conducts tetanus case surveillance through the National Notifiable Diseases Surveillance System (NNDSS) (*7*). After health care providers report tetanus cases to their local or state health departments, health departments report cases to CDC using the Council of State and Territorial Epidemiologists’ 2010 case definition.* Tetanus is a clinical diagnosis; no confirmatory laboratory tests and no case definition for confirmed tetanus exist. Probable cases are those with either 1) an acute illness with muscle spasms or hypertonia and a diagnosis of tetanus by a health care provider in the absence of a more likely diagnosis, or 2) death, with tetanus listed on the death certificate as the cause of death or as a significant condition contributing to death (*8*). Data collected through NNDSS include patients’ demographic characteristics; clinical history, including wound management and vaccination status; tetanus clinical type (cephalic, generalized, or localized); clinical management; and vital status. CDC conducted a review of data reported through NNDSS for completeness and contacted state health departments to request any additional information available to improve completeness. This activity was reviewed by CDC, deemed not research, and was conducted consistent with applicable Federal law and CDC policy.^†^

### Analytic Methods

The incidence of tetanus cases and tetanus deaths per 1 million population were calculated by year, sex, age group, and U.S. Health and Human Services (HHS) geographic region using National Center for Health Statistics’ 2009–2020 bridged-race population estimates and U.S. Census Bureau 2021–2023 single-race population estimates^§^ (*9**,**10*). Vital status by patient characteristics was examined, and case-fatality rates (CFRs) were calculated. A sensitivity analysis of CFR was conducted to assess its variability under three scenarios for patients with unknown vital status: 1) all survived, 2) none survived, and 3) one half survived. Among patients with acute wounds, clinical presentation and management (administration of tetanus immune globulin [TIG] and tetanus toxoid–containing vaccine [TTCV]) were characterized. A summary of clinical wound management to prevent tetanus is provided (Box). For this analysis, wounds were classified as tetanus-prone if they met any of the following criteria: 1) presence of devitalized tissue, 2) signs of infection or contamination, 3) puncture or crush injuries, 4) avulsions, 5) compound fractures, or 6) depth >1 cm. Persons were considered eligible for both TIG and a TTCV dose if they had a tetanus-prone wound and a history of receipt of <3 TTCV doses. In addition, persons were considered eligible for TTCV if the wound was tetanus-prone and ≥5 years had elapsed since their most recent TTCV dose or if the wound was not tetanus-prone but ≥10 years had elapsed since their most recent TTCV dose. All included proportions are among patients with known information for each data element unless otherwise stated. Data management, analyses, and visualizations were conducted using R software (version 4.4.3; R Foundation).

BOX **Wound management guidelines to prevent tetanus**

**Evaluate wound**

Dirty or major wounds include

- Penetrating or puncture wound
- Wound containing dirt, soil, feces, or saliva (e.g., animal or human bites)
- Wound containing devitalized tissue, including
  - Burns
  - Compound fractures
  - Crush injuries
  - Frostbite
  - Wounds with necrosis or gangrene

Clean and minor wounds include all other wounds.

**Provide wound care**

- Clean wound thoroughly.
- Remove dirt or foreign material and debride necrotic tissue.
- Treat infection if present.*

**Tetanus toxoid–containing vaccine (TTCV) prophylaxis recommendations**

TTCV for all wound types

- Indicated when vaccination history is unknown
- Indicated when patient is unvaccinated
- Indicated when primary series is incomplete^†^

TTCV for dirty or major wounds

- Not indicated when primary series complete and <5 years since last dose
- Indicated when primary series complete and ≥5 years since last dose

TTCV for clean and minor wounds

- Not indicated when primary series complete and <10 years since last dose
- Indicated when primary series complete and ≥10 years since last dose

**Tetanus immune globulin (TIG) prophylaxis recommendations**

TIG for dirty or major wounds

- Indicated when vaccination history is unknown^§^
- Indicated when patient has never received vaccine^§^
- Indicated when primary vaccination series is incomplete^§^
- Indicated when patient has HIV infection or severe immunodeficiency, regardless of TTCV history^§^
- Not indicated when primary series is complete^†^

TIG is not indicated for clean and minor wounds regardless of vaccination status.

* Antibiotics (topical or systemic) are not recommended to protect against tetanus during wound care.

^†^ A total of ≥3 appropriately spaced doses of a TTCV-containing vaccine are needed to have completed the primary series.

^§^ If indicated, administer 250 international units of TIG intramuscularly. TIG is commercially available for purchase. CDC does not stockpile or supply TIG.

## Results

During 2009–2023, a total of 402 cases of tetanus were reported from 47 states and the District of Columbia, and 37 tetanus deaths were reported from 16 states. A mean of 26.8 cases (range = 17–37 cases [SD = 6.3]) and 2.5 deaths (range = 0–5 deaths [SD = 1.7]) were reported each year. Many case reports had missing data, including vital status (26%), wound characteristics and management (8%–73%), and risk factors, including vaccination history (up to 80%). 

### Incidence

**FIGURE 1:**
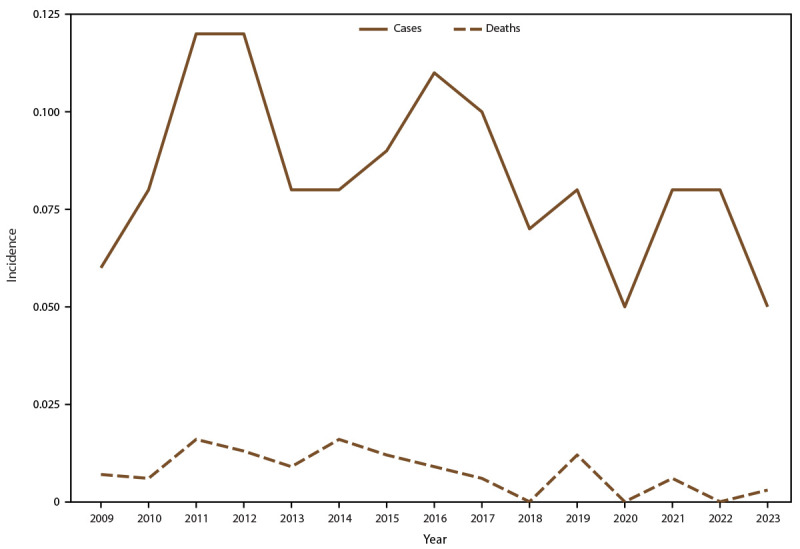
Annual incidence* of tetanus cases and tetanus-associated deaths — National Notifiable Diseases Surveillance System, United States, 2009−2023 * Per 1 million population, calculated from the National Center for Health Statistics’ 2009–2020 bridged-race population estimates (https://www.cdc.gov/nchs/nvss/bridged_race.htm) and U.S. Census Bureau (https://data.census.gov) 2021–2023 single-race population estimates.

**FIGURE 2:**
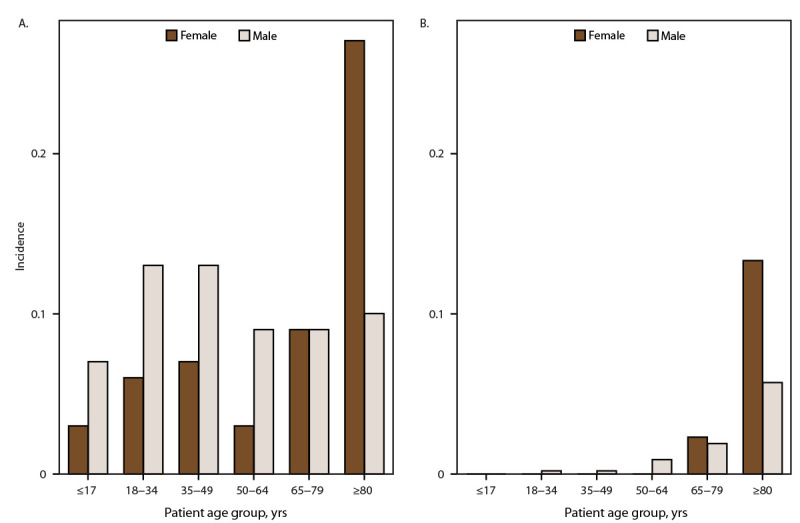
Incidence* of tetanus cases (A) and tetanus-associated deaths (B), by age group and sex — National Notifiable Diseases Surveillance System, United States, 2009–2023 * Per 1 million population, calculated from the National Center for Health Statistics’ 2009–2020 bridged-race population estimates (https://www.cdc.gov/nchs/nvss/bridged_race.htm) and U.S. Census Bureau (https://data.census.gov) 2021–2023 single-race population estimates.

The mean annual tetanus incidence was 0.08 cases per 1 million population (Table 1) (Figure 1). A total of 250 (62.2%) tetanus patients were male (Table 2), with an incidence of 0.1 per 1 million population among males compared with 0.06 among females (Table 1). The mean age of patients with tetanus was 43 years (SD = 24) and was higher among females (50 years [SD = 26]) compared with males (38 years [SD = 21]). Tetanus incidence was highest among adults aged ≥80 years (0.20 cases per 1 million population), and lowest among children and adolescents aged ≤17 years (0.05 cases per 1 million population) (Table 1) (Figure 2). Stratified by sex and age group, incidence was higher among males compared with females among all age groups for persons aged <65 years and higher among women (0.27) compared with men (0.10) among those aged ≥80 years. Tetanus incidence was highest among persons in HHS Regions 4 and 7 (0.11 each) and lowest among those in HHS Region 2 (0.04) (Table 2).

**TABLE 1:** Incidence of tetanus cases and deaths, by year, sex, age group, and U.S. Health and Human Services region* — National Notifiable Diseases Surveillance System, United States, 2009–2023

Characteristic	No. of tetanus cases	Population	Case incidence^†^ per 1 million	No. of tetanus deaths	Death incidence per 1 million
**Year**					
2009	18	306,771,529	0.06	2	0.007
2010	26	309,327,143	0.08	2	0.006
2011	36	311,583,481	0.12	5	0.016
2012	37	313,877,662	0.12	4	0.013
2013	26	316,059,947	0.08	3	0.009
2014	25	318,386,329	0.08	5	0.016
2015	29	320,738,994	0.09	4	0.012
2016	34	323,071,755	0.11	3	0.009
2017	33	325,122,128	0.10	2	0.006
2018	23	326,838,199	0.07	0	—
2019	26	328,329,953	0.08	4	0.012
2020	17	329,484,123	0.05	0	—
2021	28	332,048,977	0.08	2	0.006
2022	26	333,271,411	0.08	0	—
2023	18	334,914,895	0.05	1	0.003
**Sex**					
Male	250	2,379,850,868	0.10	15	0.0063
Female	152	2,449,975,658	0.06	22	0.0090
**Age group, yrs**					
≤17	56	1,103,040,789	0.05	0	—
18–34	108	1,121,781,180	0.10	1	0.001
35–49	94	937,149,891	0.10	1	0.001
50–64	57	930,200,814	0.06	4	0.004
65–79	50	554,916,589	0.09	12	0.022
≥80	37	182,737,263	0.20	19	0.104
**Sex and age group, yrs**					
Female ≤17	16	539,295,723	0.03	0	—
Male ≤17	40	563,745,066	0.07	0	—
Female 18–34	33	551,818,359	0.06	0	—
Male 18–34	75	569,962,821	0.13	1	0.002
Female 35–49	31	470,475,664	0.07	0	—
Male 35–49	63	466,674,227	0.13	1	0.002
Female 50–64	14	477,249,453	0.03	0	—
Male 50–64	43	452,951,361	0.09	4	0.009
Female 65–79	28	298,289,821	0.09	7	0.023
Male 65–79	22	256,626,768	0.09	5	0.019
Female ≥80	30	112,846,638	0.27	15	0.133
Male ≥80	7	69,890,625	0.10	4	0.057
U.S. Health and Human Services region*					
1	10	221,587,714	0.05	2	0.009
2	15	427,464,341	0.04	0	—
3	34	459,398,456	0.07	4	0.009
4	104	974,217,547	0.11	12	0.012
5	77	784,785,271	0.10	6	0.008
6	49	619,958,902	0.08	5	0.008
7	24	210,281,808	0.11	0	—
8	17	176,676,566	0.10	0	—
9	46	748,541,154	0.06	7	0.009
10	26	206,914,767	0.13	1	0.005
**Total**	**402**	**4,829,826,526^§^**	**0.08**	**37**	**0.008**

* U.S. Health and Human Services regions.

^†^ Per 1 million population, calculated from the National Center for Health Statistics’ 2009–2020 bridged-race population estimates (https://www.cdc.gov/nchs/nvss/bridged_race.htm) and U.S. Census Bureau (https://data.census.gov) 2021–2023 single-race population estimates.

^§^ Sum of total U.S. population from 2009–2023.

**TABLE 2:** Demographic and clinical characteristics among persons with tetanus, by vital status and case-fatality rates — United States, 2009–2023

Characteristic	Overall	Known vital status	Deaths	Case-fatality rate
	No. (%)	No. (%)	No. (%)	%
**Age group**				
<28 days (neonatal)	3 (0.7)	3 (1.0)	0 (—)	—
≥28 days to ≤17 yrs	53 (13.2)	35 (11.7)	0 (—)	—
18–34 yrs	108 (26.9)	83 (27.8)	1 (2.7)	1.2
35–49 yrs	94 (23.4)	65 (21.7)	1 (2.7)	1.5
50–64 yrs	57 (14.2)	44 (14.7)	4 (10.8)	9.1
65–79 yrs	50 (12.4)	39 (13.0)	12 (32.4)	30.8
≥80 yrs	37 (9.2)	30 (10.0)	19 (51.4)	63.3
**Sex**				
Female	152 (37.8)	118 (39.5)	22 (59.5)	18.6
Male	250 (62.2)	181 (60.5)	15 (40.5)	8.3
**Sex and age group**				
Female <28 days (neonatal)	1 (0.2)	1 (0.3)	0 (—)	—
Male <28 days (neonatal)	2 (0.5)	2 (0.7)	0 (—)	—
Female ≥28 days to ≤17 yrs	15 (3.7)	10 (3.3)	0 (—)	—
Male ≥28 days to ≤17 yrs	38 (9.5)	25 (8.4)	0 (—)	—
Female 18–34 yrs	33 (8.2)	25 (8.4)	0 (—)	—
Male 18–34 yrs	75 (18.7)	58 (19.4)	1 (2.7)	1.7
Female 35–49 yrs	31 (7.7)	24 (8.0)	0 (—)	—
Male 35–49 yrs	63 (15.7)	41 (13.7)	1 (2.7)	2.4
Female 50–64 yrs	14 (3.5)	10 (3.3)	0 (—)	—
Male 50–64 yrs	43 (10.7)	34 (11.4)	4 (10.8)	11.8
Female 65–79 yrs	28 (7.0)	23 (7.7)	7 (18.9)	30.4
Male 65–79 yrs	22 (5.5)	16 (5.4)	5 (13.5)	31.2
Female ≥80 yrs	30 (7.5)	25 (8.4)	15 (40.5)	60.0
Male ≥80 yrs	7 (1.7)	5 (1.7)	4 (10.8)	80.0
**Race**				
Asian or Pacific Islander	7 (2.0)	7 (2.6)	1 (2.9)	14.3
Black or African American	40 (11.4)	24 (9.0)	2 (5.9)	8.3
Native American or Alaska Native	2 (0.6)	2 (0.7)	0 (—)	—
White	279 (79.3)	214 (79.9)	29 (85.3)	13.6
Other	24 (6.8)	21 (7.8)	2 (5.9)	9.5
Unknown	50 (—*)	31 (—)	3 (—)	9.7
**Ethnicity**				
Hispanic or Latino	44 (13.3)	38 (14.8)	6 (20.7)	15.8
Non-Hispanic or Latino	286 (86.7)	219 (85.2)	23 (79.3)	10.5
Unknown	72 (—)	40 (—)	8 (—)	20.0
**Diabetes**				
No	256 (88.6)	214 (89.2)	21 (70.0)	9.8
Yes	33 (11.4)	26 (10.8)	9 (30.0)	34.6
Unknown^†^	113 (—)	56 (—)	7 (—)	12.5
**Intravenous drug use**				
No	222 (89.2)	185 (90.2)	24 (92.3)	13.0
Yes	27 (10.8)	20 (9.8)	2 (7.7)	10.0
Unknown^†^	153 (—)	94 (—)	11 (—)	11.7
**TTCV dose history**				
Number of TTCV doses				
None	76 (43.9)	64 (41.6)	8 (50.0)	12.5
1–2 doses	58 (33.5)	53 (34.4)	8 (50.0)	15.1
≥3 doses	39 (22.5)	37 (24.0)	0 (—)	—
Unknown^†^	229 (—)	145 (—)	21 (—)	14.5
TTCV received <10 yrs previously^§^				
Yes	33 (41.3)	31 (41.3)	2 (33.3)	6.5
No	47 (58.8)	44 (58.7)	4 (67.7)	9.1
Unknown^†^	322 (—)	224 (—)	31 (—)	13.9
**Wound characteristics**				
Acute wound				
No	47 (13.2)	38 (13.2)	2 (5.9)	5.3
Yes	310 (86.8)	249 (86.8)	32 (94.1)	12.9
Unknown^†^	45 (—)	12 (—)	3 (—)	25.0
Work-related wound				
No	141 (84.9)	116 (84.1)	22 (100.0)	19.0
Yes	25 (15.1)	22 (15.9)	0 (—)	—
Unknown or no acute wound^†,¶^	236 (—)	161 (—)	15 (—)	9.3
Anatomic site				
Head	19 (6.6)	14 (6.1)	2 (6.9)	14.3
Trunk	6 (2.1)	6 (2.6)	0 (—)	—
Upper extremity	108 (37.8)	88 (38.3)	11 (37.9)	12.5
Lower extremity	153 (53.5)	122 (53.0)	16 (55.2)	13.1
Unknown or no acute wound^†,¶^	116 (—)	69 (—)	8 (—)	11.6
Wound type				
Puncture	158 (61.2)	125 (59.8)	8 (34.8)	6.4
Stellate or linear laceration	51 (19.8)	44 (21.1)	5 (21.7)	11.4
Abrasion	35 (13.6)	30 (14.4)	8 (34.8)	26.7
Crush, avulsion, burn, or compound fracture	14 (5.4)	10 (4.8)	2 (8.7)	20.0
Unknown or no acute wound^†,¶^	144 (—)	90 (—)	14 (—)	15.6
Tetanus-prone wound**				
No	4 (1.8)	4 (2.3)	1 (4.8)	25.0
Yes	214 (98.2)	171 (97.7)	20 (95.2)	11.7
Unknown or no acute wound^†,¶^	184 (—)	124 (—)	16 (—)	12.9
**Clinical care**				
Medical care obtained for acute injury				
No	88 (55.0)	67 (51.9)	8 (42.1)	11.9
Yes	72 (45.0)	62 (48.1)	11 (57.9)	17.7
Unknown or no acute wound^†,¶^	242 (—)	170 (—)	18 (—)	10.6
Wound debrided before tetanus onset				
No	62 (74.7)	52 (75.4)	9 (90.0)	17.3
Yes	21 (25.3)	17 (24.6)	1 (10.0)	5.9
Unknown or no acute wound^†,¶^	319 (—)	230 (—)	27 (—)	11.7
**Post-exposure prophylaxis **				
TIG prophylaxis indicated after acute injury^††^				
No	26 (23.6)	25 (25.8)	0 (—)	—
Yes	84 (76.4)	72 (74.2)	10 (100)	13.9
Unknown or no acute wound^†,¶^	292 (—)	202 (—)	27 (—)	13.4
TIG prophylaxis administered before tetanus onset				
No	132 (87.4)	110 (88.7)	17 (94.4)	15.5
Yes	19 (12.6)	14 (11.3)	1 (5.6)	7.1
Unknown or no acute wound^†,¶^	251 (—)	175 (—)	19 (—)	10.9
TIG prophylaxis indicated and administered before tetanus onset				
No	43 (97.7)	38 (97.4)	6 (100)	15.8
Yes	1 (2.3)	1 (2.6)	0 (—)	—
Unknown or no acute wound^†,¶^	358 (—)	260 (—)	31 (—)	11.9
TTCV indicated after acute injury^††^				
No	3 (2.8)	3 (3.2)	0 (—)	—
Yes	103 (97.2)	90 (96.8)	10 (100)	11.1
Unknown	296 (—)	206 (—)	27 (—)	13.1
TTCV administered before tetanus onset but after acute injury				
No	84 (74.3)	69 (73.4)	9 (75.0)	13.0
Yes	29 (25.7)	25 (26.6)	3 (25.0)	12.0
Unknown or no acute wound^†,¶^	289 (—)	205 (—)	25 (—)	12.2
TTCV indicated and administered before tetanus onset but after acute injury				
Yes	12 (28.6)	12 (31.6)	1 (25.0)	8.3
Unknown or no acute wound^†,¶^	360 (—)	261 (—)	33 (—)	12.6
**Type of tetanus**				
Generalized	113 (74.8)	91 (72.8)	11 (64.7)	12.1
Localized	35 (23.2)	31 (24.8)	4 (23.5)	12.9
Cephalic	3 (2.0)	3 (2.4)	2 (11.8)	66.7
Unknown	251 (—)	174 (—)	20 (—)	11.5
**Outcome**				
Hospitalized				
No	12 (5.1)	10 (5.0)	0 (—)	—
Yes	225 (94.9)	192 (95.0)	30 (100)	15.6
Unknown	165 (—)	97 (—)	7 (—)	7.2
Admitted to intensive care unit				
No	76 (35.0)	67 (35.0)	2 (7.4)	3.0
Yes	141 (65.0)	122 (65.0)	25 (92.6)	20.5
Unknown	185 (—)	110 (—)	10 (—)	9.1
Treated with mechanical ventilation				
No	126 (58.1)	109 (58.0)	6 (22.2)	5.5
Yes	91 (41.9)	80 (42.0)	21 (77.8)	26.2
Unknown	185 (—)	110 (—)	10 (—)	9.1
**Total**	**402**	**299**	**37**	**12.4**

**Abbreviations:** TIG = tetanus immune globulin; TTCV = tetanus toxoid–containing vaccine.

* Unknown entries do not include column percentages because calculations were performed among cases with known data for each element.

^†^ Includes three neonates with cases of tetanus for whom this variable was not applicable.

^§^ Among persons with ≥1 documented TTCV dose.

^¶^ Includes persons without a documented acute wound.

** Wounds were classified as tetanus-prone if they met any of the following criteria: presence of devitalized tissue, signs of infection or contamination, puncture or crush injuries, avulsions, compound fractures, or depth >1 cm.

^††^ Persons were considered eligible for both TIG and a TTCV dose if they had a tetanus-prone wound and a history of <3 TTCV doses. In addition, persons were considered eligible for TTCV if the wound was tetanus-prone and >5 years had elapsed since their most recent TTCV dose or if the wound was not tetanus-prone but ≥10 years had elapsed since their most recent TTCV dose.

### Vaccination and Clinical Care

Seventy-six of 173 (43.9%) tetanus cases were among persons who had no documented history of receiving a TTCV dose. Among 80 persons with tetanus who had received at least 1 TTCV dose, 47 (58.8%) received their most recent TTCV dose ≥10 years before tetanus onset (Table 2); the median interval since the most recent TTCV dose was 12.5 years (IQR = 7–20 years). Generalized tetanus was the most common clinical type (113 of 151 [74.8%]), followed by localized (35 of 151 [23.2%]) and cephalic (3 of 151 [2.0%]). A total of 225 of 237 (94.9%) persons were hospitalized; of those hospitalized, 65.0% (141 of 217) required care in an intensive care unit and 41.9% (91 of 217) required mechanical ventilation.

### Neonatal Tetanus

Three cases of tetanus were identified among neonates, all of whom survived. Of the two neonates with available information, both births occurred in a home setting with a midwife, and neither mother had documentation of ever having received a TTCV dose.

### Wound Management

Most tetanus cases (310 of 357 [86.8%]) were associated with an acute wound (Table 2). A majority of wounds were punctures (158 of 258 [61.2%]) or stellate or linear lacerations (51 of 258 [19.8%]) and were located on an extremity (261 of 286 [91.3%]). Nearly all (214 of 218 [98.2%]) wounds were classified as tetanus-prone. Among persons with wounds, 72 of 160 (45.0%) sought medical care for their wound before tetanus onset. TTCV prophylaxis was administered to a minority (12 of 42 [28.6%]) of patients for whom TTCV was indicated. TIG prophylaxis was administered for one of 44 (2.3%) patients with wounds for whom TIG was indicated.

### Mortality and Case-Fatality Rates

The mean annual tetanus-associated mortality rate was 0.008 deaths per 1 million population (Table 1) (Figure 1). Among the 402 reported tetanus cases, vital status was known for 299 (74.4%). Among female patients, 22 of 118 (18.6%) died; among male patients, 15 of 181 (8.3%) died (Table 2) (mortality rate = 0.009 deaths per 1 million population among females and 0.006 among males) (Table 1). The median age at death for both sexes was 80 years (IQR = 73–86 years). Tetanus-associated deaths occurred at a younger median age among males (70 years; minimum age = 26 years) than among females (median age = 84 years; minimum age = 72 years). Tetanus-associated mortality was highest among adults aged ≥80 years (0.10 deaths per 1 million population); no deaths were reported among children and adolescents aged ≤17 years (Table 2). Among adults aged 65–79 years, the mortality rate was comparable among women (0.023 per 1 million population) and men (0.019), but among adults aged ≥80 years, mortality was twice as high among women (0.133) as it was among men (0.057) (Table 1) (Figure 2). The reported tetanus mortality rate was highest in HHS Region 4 (0.012); no deaths were reported by HHS Regions 2, 7, or 8.

Among persons with tetanus whose vital status was known, the CFR was 12.4% (37 of 299) (Table 2). In sensitivity analyses, the CFR was 9.2% (37 of 402) assuming that all persons with unknown vital status survived; 22.0% (89 of 402) assuming that one half survived; and 34.8% (140 of 402) assuming that all persons with unknown vital status died. Among those with known vital status, the CFR was higher among females (18.6%) than among males (8.3%). A majority (83.8%) of deaths occurred among those aged ≥65 years. (The CFR among adults aged 65–79 and ≥80 years were 30.8% and 63.3%, respectively.) When stratified by age group, CFRs among males and females were comparable. The CFR was higher among persons with cephalic tetanus (66.7%) than among those with generalized tetanus (12.1%). The median interval from symptom onset until death was 13.0 days (IQR = 8.0–20.1 days).

No deaths were reported among persons with tetanus who had received ≥3 TTCV doses. Two persons with documentation of receipt of TTCV who died had received their most recent dose <10 years preceding death (1 and 7 years), but both were aged ≥80 years and had only 1 TTCV dose recorded in their vaccination history; thus, they likely had not received a full TTCV series, which is needed for adequate protection (*11*). Among the 39 persons with known vital status who had an acute wound for which TIG was indicated, one received TIG before disease onset; this person survived. Among those with known vital status with an acute wound for which TTCV was indicated, 31.6% (12 of 38) received TTCV, and one person who received TTCV before disease onset died. 

## Discussion

Tetanus remains rare in the United States, underscoring the success of current vaccination policies and programs (*11*,*12*). Incidence during 2009–2023 (0.08 cases per 1 million population) was slightly lower than that reported during the previous surveillance period of 2001–2008 (0.10 cases per 1 million); the incidence of U.S. tetanus cases during this period was >100 times lower than the estimated global incidence of 10.3 cases per 1 million in 2019 (*6*,*13*). Tetanus primarily affects adults, with 86% of all cases occurring among adults aged ≥18 years. However, the median age of tetanus patients (40 years) during 2009–2023 was lower than that during 2001–2008 (49 years) (*6*). Tetanus incidence among adults aged ≥80 years is higher than for all other age groups. This cohort was born before the primary series of tetanus toxoid vaccines was recommended for routine use in 1947. The lowest incidence among children compared with other ages likely reflects the impact of high coverage with recommended tetanus immunizations for children and adolescents: for children, routine childhood diphtheria and tetanus toxoids, and acellular pertussis (DTaP) vaccines (>92% coverage with ≥3 doses by age 35 months for those born during 2011–2021) and for adolescents, adolescent tetanus toxoid, reduced diphtheria toxoid and acellular pertussis vaccine (Tdap) boosters (>76% coverage among adolescents aged 13–17 years during 2009–2024 [>88% since 2016]) (*14*,*15*). Adult coverage with a combined tetanus and diphtheria toxoid vaccine or Tdap is substantially lower (57%–70% during 2013–2022) than that among children or adolescents (*16*). Although TTCVs are recommended in childhood and as decennial boosters throughout life, older adults might have missed opportunities for booster vaccination.

Tetanus incidence in the United States varies by age, with sex modifying this association. Overall, among persons aged <65 years, incidence among males is higher than females. However, among adults aged ≥80 years, the incidence is higher among women. The higher incidence among younger men might reflect sex differences in occupational and recreational exposures. The higher incidence among the oldest group of women compared with the oldest group of men might be due to historical sex differences in military service wherein men would have been required to receive tetanus toxoid vaccines (*17*), or to persistence of antibodies among men; findings from a national serosurvey of tetanus reported lower seroprotection among older women compared with men (*18*). Sex differences might be also due to variations in wound frequency and clinical wound management. However, tetanus incidence worldwide remains higher among males than females throughout the lifespan (*13*).

Tetanus is a serious and life-threatening disease. Ninety-five percent of patients with reported tetanus are hospitalized. Approximately 1 in 10 persons who develop tetanus in the United States will die. The risk for death increases with increasing age: 85% of tetanus deaths occurred among adults aged ≥65 years. The higher incidence of death among females than among males is likely driven by the higher overall incidence of tetanus among women in the oldest age groups compared with men. Although females experience higher tetanus-associated mortality, the CFR stratified by age is similar among males and females, indicating that the risk for death among males and females who contract tetanus is similar.

Although neonatal tetanus is extraordinarily rare in the United States, cases still occur. Among neonates with known birth information, in the two cases where deliveries occurred at home, the lack of maternal TTCV vaccination combined with nonsterile delivery setting likely contributed to the development of neonatal tetanus. Worldwide, particularly in low-income countries, neonatal tetanus remains a substantial cause of morbidity and mortality after unhygienic deliveries in settings with low TTCV coverage and lacking skilled birth attendants; approximately 8,000 neonatal tetanus cases were reported worldwide in 2021 (*19**,**20*).

Tetanus and tetanus deaths are preventable through routine vaccination and recommended wound care and management (*21*). Approximately one half of patients with tetanus had no documented history of ever having received a TTCV, and the majority who had received ≥1 TTCV dose most recently received it ≥10 years before tetanus onset. No deaths were reported among patients with documented receipt of ≥3 TTCV doses. The two persons who died who had received a TTCV in the preceding 10 years did not have records indicating completion of their primary series, underscoring the importance of completing the primary series, in addition to decennial boosters, for protection. These findings are consistent with previous studies that reported less favorable outcomes among persons with tetanus who failed to complete the full childhood vaccination series or remain up-to-date (*22*,*23*). Although the large majority of persons with tetanus who had wounds had a tetanus-prone wound, fewer than one half sought medical care before disease onset. TTCV was indicated for nearly all patients, and TIG was indicated for approximately 75%. However, only approximately one third received TTCV, and one in 50 received TIG. Among those whose vital status was known, no patient died who received TIG prophylaxis, and one patient who received TTCV prophylaxis died, highlighting the importance of receipt of prompt and recommended treatment to reduce the risk for death.

Despite the rarity of tetanus, vigilance among clinicians is critical, and providers might benefit from continuing education regarding the indications for TTCV and TIG in wound management. These findings are consistent with those in the 2001–2008 surveillance report, which indicated that further education and support are needed for persons with wounds to ensure that they seek care. In addition, health care providers should ensure that their patients’ TTCV doses are up-to-date and follow treatment guidelines to determine the need for TIG and TTCV (*24*). For patients with tetanus, receiving all recommended TTCV doses during recovery is critical because contracting and surviving tetanus does not confer immunity to future infection (*1*).

## Limitations

The findings in this report are subject to at least two limitations. First, tetanus surveillance is passive, and cases might be underreported; however, because tetanus is a serious disease requiring hospitalization, the underestimation is likely minimal. Second, many case reports had missing data, which might introduce bias if the cases for which data elements are reported or are missing are different; data absence was minimized by requesting key data elements directly from jurisdictions.

## Future Directions

These findings can support clinicians in communicating the importance of administering TTCV (as DTaP) to children as part of the routine childhood DTaP vaccination schedule, as well as decennial tetanus boosters (as Td or Tdap). Tetanus boosters are particularly important for older adults who might not have received the primary series or who might not be current with their adult booster dose, and for pregnant women whose infants are at risk for neonatal tetanus if their mothers are not fully vaccinated. Cleaning wounds with soap and water after an injury and timely and recommended wound care by clinicians is important to identify the potential need for TTCV or TIG administration. Understanding the barriers to receipt of recommended wound management and improving provider education about the importance of wound management are critical. The increased tetanus incidence and associated deaths among older age groups, especially women, suggest waning of vaccine-induced immunity and underscore the need for focused vaccination and wound care prevention efforts among these groups. Comprehensive tetanus case reporting by local and state public health officials is essential to monitoring future trends and identifying opportunities for tetanus prevention.

## Conclusion

*C. tetani* bacteria are ubiquitous in the environment, and despite being preventable, tetanus remains a risk for those who are unvaccinated, undervaccinated or who do not receive recommended wound management. Tetanus incidence varies by age and sex. Overall incidence is higher in males, but for those aged ≥80 years, rates are higher in females. Approximately one in 10 persons who develop tetanus, even with treatment, will die, with the highest mortality among older adults. Sustained routine vaccination with TTCV and prompt identification and management of tetanus-prone injuries are essential to preserving the current low incidence of this potentially fatal disease.

## Footnotes

* The definition used in 2009 was functionally the same but included only a confirmed definition and no mention of tetanus as a cause of death. The 1996 definition of a confirmed case (used through 2009) was a clinically compatible case, as reported by a health care professional.

† 45 C.F.R. part 46.102(l)(2), 21 C.F.R. part 56; 42 U.S.C. Sect. 241(d); 5 U.S.C. Sect. 552a; 44 U.S.C. Sect. 3501 et seq.

§ CDC WONDER U.S. bridged-race July 1st resident population estimates by state, county, age, sex, bridged-race, and Hispanic or Latino origin; compiled from revised bridged-race 2000–2009 intercensal population estimates (Documentation for Revised 2000–2009 Bridged-Race Intercensal Population Estimates for Calculating Vital Rates | NCHS) and bridged-race vintage 2020 (2010–2020) postcensal population estimates (Documentation for Vintage 2020 Bridged-Race Postcensal Population Estimates for Calculating Vital Rates | NCHS).
